# Interaction between Aβ and Tau in the Pathogenesis of Alzheimer's Disease

**DOI:** 10.7150/ijbs.57078

**Published:** 2021-05-27

**Authors:** Huiqin Zhang, Wei Wei, Ming Zhao, Lina Ma, Xuefan Jiang, Hui Pei, Yu Cao, Hao Li

**Affiliations:** 1 Institute of Geriatrics, Xiyuan Hospital, China Academy of Chinese Medical Sciences, Beijing 100091, China.; 2 Beijing University of Chinese Medicine, Beijing 100029, China.

**Keywords:** Alzheimer's disease, amyloid-β, tau, interaction, phosphorylation

## Abstract

Extracellular neuritic plaques composed of amyloid‑β (Aβ) protein and intracellular neurofibrillary tangles containing phosphorylated tau protein are the two hallmark proteins of Alzheimer's disease (AD), and the separate neurotoxicity of these proteins in AD has been extensively studied. However, interventions that target Aβ or tau individually have not yielded substantial breakthroughs. The interest in the interactions between Aβ and tau in AD is increasing, but related drug investigations are in their infancy. This review discusses how Aβ accelerates tau phosphorylation and the possible mechanisms and pathways by which tau mediates Aβ toxicity. This review also describes the possible synergistic effects between Aβ and tau on microglial cells and astrocytes. Studies suggest that the coexistence of Aβ plaques and phosphorylated tau is related to the mechanism by which Aβ facilitates the propagation of tau aggregation in neuritic plaques. The interactions between Aβ and tau mediate cognitive dysfunction in patients with AD. In summary, this review summarizes recent data on the interplay between Aβ and tau to promote a better understanding of the roles of these proteins in the pathological process of AD and provide new insights into interventions against AD.

## Introduction

Alzheimer's disease (AD) is a progressive neurodegenerative disease that is characterized by the progressive deterioration of cognition and memory 1. AD is defined pathologically by an extensive loss of neurons and two characteristic protein deposits in the brain, extracellular amyloid plaques and intracellular neurofibrillary tangles (NFTs), which are caused by tau hyperphosphorylation 1, 2. Several hypotheses related to the pathogenesis of AD were studied 3, and the amyloid-β (Aβ) cascade and the hyperphosphorylation of tau protein are the two main hypotheses. The Aβ cascade hypothesis states that Aβ, deposited in the form of neuroinflammatory plaques, induces AD by damaging neuronal cells 4. Aβ facilitates the development of AD and initiates a deleterious cascade involving tau pathology and neurodegeneration 5. However, the development of many AD drugs examined in clinical trials and laboratory studies focused on the reduction of amyloid deposition and the clearance of Aβ oligomers and have not yielded satisfactory effects 6-8. Aβ may not be the only disease-causing protein during the course of AD. Intraneuronal tangles containing hyperphosphorylated tau are a hallmark of AD pathology 9, and tau is a mediator of Aβ cytotoxicity 7. Therefore, the pathological changes to tau in AD also attracted attention. However, extensive studies focused solely on the neurotoxicity of Aβ or tau have not shown significant efficacy in the treatment of AD 8-11. Therefore, focusing solely on the role of Aβ or tau in AD lesions while ignoring the interaction between Aβ and tau may not be entirely correct. Aβ and tau may interact via intermediate materials, such as kinases (e.g., GSK-3β, CDK-5 and ERK) 12. The removal of Aβ or tau alone does not completely terminate the interaction pathway, which continues to play a role in the acceleration of the pathological process 11, 13. The pathological process likely involves an interplay between Aβ and tau in which these proteins amplify toxic effects rather than a strictly hierarchical mode of interaction 13. Therefore, suppression of the interaction may be of more practical significance than simply focusing on the neurotoxicity of Aβ or tau alone 14. The present review describes the following interactions between Aβ and tau. A) Aβ drives tau pathology by inducing tau hyperphosphorylation, and hyperphosphorylated tau mediates toxicity in neurons. B) Tau mediates Aβ toxicity, and Aβ toxicity is critically dependent on the presence of tau. C) Aβ and tau target cellular processes or organelles synergistically and may amplify each other's toxic effects. D) Aβ and tau may coexist in pathological locations 11. We hope that this review provides a better understanding of the role of Aβ and tau in the pathological development of AD to promote further research on the mechanism of the interaction between Aβ and tau and the development of more effective therapies for AD.

## Aβ mediates tau toxicity

### Aβ accelerates the phosphorylation of tau protein

**Figure 1:**
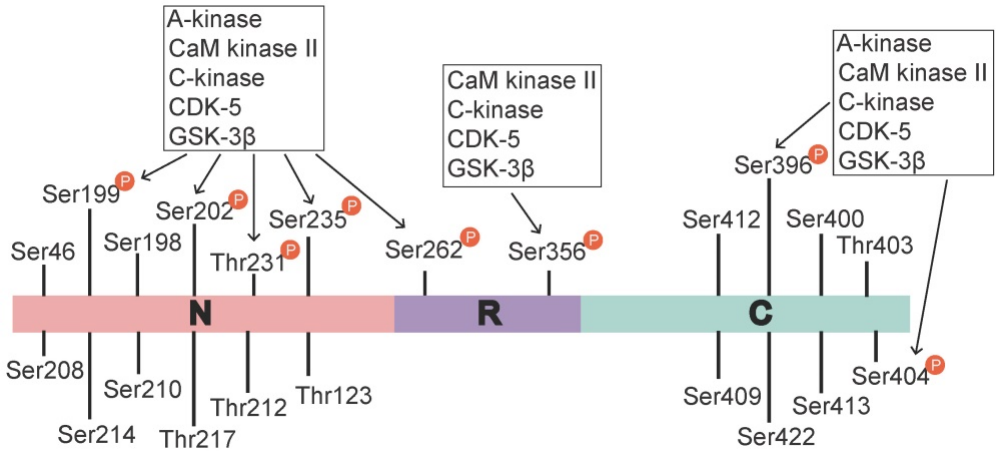
** Tau phosphorylation sites and associated kinases.** The identified tau phosphorylation sites are located in the N-terminal region (Ser46 16, Thr123 17, Ser198, Ser199, Ser202, Ser208, Ser210, Thr212, Ser214, Thr217, Thr231, and Ser235), the repeat region (Ser262 and Ser356), and the C-terminal region (Ser396, Ser400, Thr403, Ser404, Ser409, Ser412, Ser413, and Ser422 18). Ser199, Ser202, Thr231, Ser235, Ser262, Ser396, and Ser404 are phosphorylated by various activated kinases, namely A-kinase, C-kinase, CaM kinase II, CDK-5 and Gsk-3β, and Ser356 is phosphorylated by C-kinase, CaM kinase II, CDK-5 and Gsk-3β. A-kinase, C-kinase, CaM kinase II and CDK-5 are primarily involved in the prephosphorylation of tau. This prephosphorylation increases the affinity of GSK-3β to these sites, which ultimately leads to tau hyperphosphorylation.

Tau is a microtubule (MT)-binding protein, and tau phosphorylation at multiple sites controls its binding to MTs 15. The identified phosphorylation sites are located in the N-terminal region (Ser46 16, Thr123 17, Ser198, Ser199, Ser202, Ser208, Ser210, Thr212, Ser214, Thr217, Thr231, and Ser235), the repeat region (Ser262 and Ser356), and the C-terminal region (Ser396, Ser400, Thr403, Ser404, Ser409, Ser412, Ser413, and Ser422 18) (Figure 1). These sites are phosphorylated by several enzymes, including A-kinase, C-kinase, cyclin-dependent kinase-5 (CDK-5), CaM kinase II, glycogen synthase kinase-3β (GSK-3β) and MAPKs 12, 19 (Table 1). Under pathological conditions, these kinases contribute to the hyperphosphorylation of tau protein, which subsequently leads to the dissociation of tau protein from MTs and the formation of NFTs 20, 21. Hyperphosphorylated tau protein results in abnormal increases in cytoskeletal proteins, axoplasmic transport disorders and neuronal degeneration 22. Tau hyperphosphorylation is the product of deregulated Ser/Thr kinases, such as CDK-5 and GSK-3β 23. Tau protein is prephosphorylated by A-kinase, C-kinase, CDK-5, and CaM kinase II and subsequently phosphorylated by GSK-3β. Ser262/Ser356 is first prephosphorylated by CDK-5, CaM kinase II and C-kinase, and this prephosphorylation makes it more accessible to GSK-3β for further phosphorylation 24. The prephosphorylation of Ser262/Ser356 may cause tau to assume a more favourable conformation such that Thr231, Ser235, Ser396, Ser404 19, 24 and other sites may be rapidly recognized by GSK-3β in the subsequent phosphorylation step 25. Aβ accelerates the hyperphosphorylation of tau by mediating the activation of CDK-5 23 and GSK-3β 26. GSK-3β, which is inextricably related to Aβ, is an important factor in the phosphorylation of tau, and it aggravates tau-induced neurotoxicity 15, 27 (Table 1). Tau phosphorylation at Ser262 is equally critical for Aβ42-induced tau toxicity 11. In conclusion, aggregated Aβ induces tau hyperphosphorylation by enhancing the activity of GSK-3β and CDK-5, which are involved in the phosphorylation of tau at multiple Ser/Thr sites 28 (Figure 1). Hyperphosphorylated tau protein detaches from MTs and is more likely to form NFTs 23, 27, 30, which causes neurofibrillary degeneration. Therefore, CDK-5 and GSK-3β kinase inhibitors may help inhibit Aβ-mediated tau hyperphosphorylation. For example, inhibitors of GSK-3β and CDK-5, such as lithium, AZD1080 and roscovitine, significantly lowered the levels of tau phosphorylation and significantly reduced the levels of aggregated, insoluble tau 31-33. However, their clinical efficacy has not been established 34. This result further suggests that proteins other than Aβ act on tau hyperphosphorylation or phosphorylation caused by kinases other than CDK-5 and GSK-3β, that Aβ is not the only protein that activates CDK-5 and GSK-3β, and that other tau phosphorylation sites should be further explored. Further studies have demonstrated that mitogen-activated protein kinases (MAPKs) and GSK-3β were involved in the formation of PHF-tau in the AD brains 35-37. Similarly, Aβ-induced tau phosphorylation in neurons is mediated via activation of both MAPK and GSK-3β 12, 36-38. MAPKs, including ERK1/2, SAPKs and p38, are activated by tyrosine phosphorylation and are involved in the phosphorylation of tau in AD 36-38. Therefore, further exploration is needed to develop new drugs that act at different specific phosphorylation sites of tau via various mechanisms.

### Aβ also interferes with tau oligomerization

**Figure 2:**
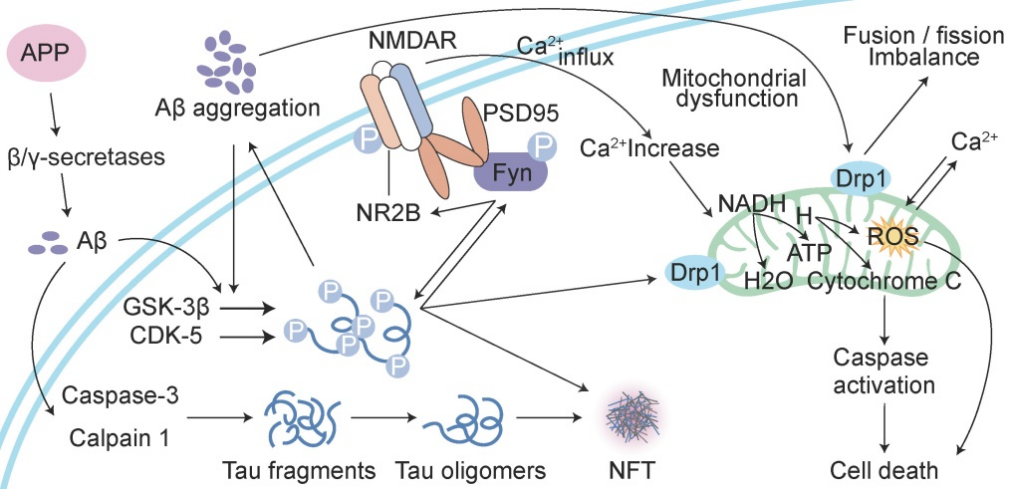
** Reciprocal toxicity between Aβ and tau.** Aβ precursor protein (APP) is cleaved by β/γ secretases to form Aβ. Aβ activates GSK-3β and CDK-5 to phosphorylate tau protein and activate caspase-3 and calpain 1 to hydrolyse tau protein and form tau oligomers. Phosphorylated tau protein interacts with Fyn. Aβ-activated Fyn also accelerates tau phosphorylation and binds to tau. Phosphorylated Fyn acts on NR2B to form the NMDAR-PSD95-Fyn complex. NMDARs are activated to increase Ca^2+^, which affects the function of mitochondria. Aβ and phosphorylated tau induce the fusion and fission of mitochondria by acting on Drp1, which induces the dysfunction of mitochondrial dynamics and ultimately leads to reactive oxygen species (ROS) overproduction and apoptosis.

In addition to its driving effect on tau phosphorylation, Aβ also interferes with tau oligomerization and aggregation. Tau oligomers, which are an intermediate form of tau that occurs prior to NFT formation, are toxic 39. Hyperphosphorylated tau dislodges from MTs, and its affinity for other tau monomers induces binding between individual tau proteins to form tau oligomers, which are detergent-soluble aggregates, by acting on the phosphorylation sites. This process is mediated by Aβ-activated CDK-5 and GSK-3β. These tau oligomers potentiate neuronal damage, which leads to neurodegeneration 40, 41. Aβ also triggers caspase-3 (CASP3) cleavage of tau at Asp421 in the C-terminus to yield an N-terminal caspase cleavage product (amino acids 1-421) 42 (Figure 2). Truncated tau lacking its C-terminal 20 amino acids assembles more rapidly into filaments than full-length tau 42. Aβ also induces the generation of a 17-kDa tau fragment via the activation of calpain-1 in hippocampal neurons. The expression of this tau fragment leads to neurite degeneration and cell death in neurons and nonneuronal cell types 43. Cleaved tau protein self-aggregates and misfolds, and these self-aggregated and misfolded products are further assembled into soluble oligomers 42. Tau oligomers may act as “seeds” by inducing endogenous tau misfolding and redistribution to the somatodendritic compartment, which suggests the existence of a unifying mechanism for the propagation of amyloid proteins 44, 45. Oligomers in this aggregation process are the most neurotoxic form of tau and may be transmitted between neuronal cells 39. Tau oligomers in neurons induce neuroinflammatory factors, which bind to astrocytes and microglia and induce apoptosis 39, 46. Therefore, the inhibition of tau oligomer formation is also worth further examination. For example, a targeted inhibitor of caspase-3 may be developed to reduce tau cleavage.

### Aβ toxicity is dependent on tau

Extracellular deposits of fibrillar Aβ form senile plaques and accelerate tau hyperphosphorylation, which induces neurotoxicity. However, whether the neurotoxicity of Aβ is dependent on tau is not clear. One study showed that the absence of tau in glutamate N-methy1-1-D-aspartate (NMDA) receptor subunit GluN2B-bearing spines, where tau is typically located, prevented the toxic effect of the binding of Aβ to NMDA receptors (NMDARs) 47. A recent study proposed that the phosphorylation of tau at Tyr 18 by Fyn kinase also blocks Aβ toxicity 48, 49. Aβ may be the initiator of tau pathology and play a role in downstream neuronal injury 50, 51. Tau mediates Aβ toxicity by interacting with Fyn kinase via its amino-terminal projection domain (PD) 50. Aβ promotes Fyn phosphorylation, and phosphorylated tau and Fyn are transferred to postsynaptic membrane receptors, which are denoted by NMDARs. Fyn phosphorylates the NR subunit 2 (NR2) to facilitate the interaction with postsynaptic density protein 95 (PSD95) 47, 52, which leads to excitotoxic downstream signalling 47, 53. Soluble Aβ binds to or near NMDARs, which indicates that NMDARs are potential targets of Aβ, and the NMDAR and PSD95 complex participates in NMDAR-mediated Aβ neurotoxicity 54, 55. Therefore, the activation of Fyn, which migrates into dendritic spines via tau facilitation, is a key event in Aβ oligomer-induced synaptic toxicity 45, 56. Some studies have also indicated that a reduction in tau levels prevented the cognitive impairment in AD transgenic mice overexpressing Aβ and that overexpression of Fyn can enhance their cognitive impairment 57, 58. The absence of tau prevents Aβ toxicity and tau phosphorylation by GSK3 59, 60 or another kinase (p38γ) 61 at a specific residue (threonine 205) 62. P38γ regulates PSD95-tau-Fyn complexes by phosphorylating tau at Thr205, which interferes with the postsynaptic excitotoxic signalling complexes engaged by Aβ. Tau-dependent Aβ toxicity is modulated by site-specific tau phosphorylation, which inhibits postsynaptic PSD95-tau-Fyn complexes, which reveals an Aβ toxicity-limiting role of p38γ in AD 61. A new study found that Aβ alone caused hyperactivity, and tau alone suppressed activity and promoted the silencing of many neurons 62. Neuronal silencing dominates hyperactivity in the presence of both Aβ and tau, and this finding was corroborated in a recent *in vitro* study using extracellular field recordings of entorhinal cortex (EC) slices 62. Tau blocks Aβ-dependent hyperactivity, which results in the profound silencing of circuits in the presence of Aβ and tau in the cortex 63. This result suggests that tau and Aβ exert antagonistic effects on neural circuit activation. Caspase-cleaved tau may also be an important downstream event in Aβ-induced cascades, such as the poisonous 17-kDa tau fragment, which leads to neurite degeneration and cell death 43. Therefore, Aβ-induced neurotoxicity may be mediated not only by Aβ overdeposition but also, at least in part, by protease activation, which leads to the generation of neurotoxic tau fragments, such as the 17-kDa neurotoxic tau fragment. Aβ may serve as the trigger, and tau may be the bullet 45, 53. All of these results further support the notion that the toxicity of Aβ is dependent on tau (Figure 2). In summary, tau protein may play a key role in the Aβ-induced mechanism, and the recombinant expression of human tau protein in tau-depleted neurons restores the sensitivity of neurons to Aβ toxicity.

### Aβ and tau work together to damage mitochondria

Another cellular compartment in which Aβ and tau work together to damage neurons is the mitochondrion 45. Mitochondrial dysfunction is widely implicated in the aetiology of AD 64. Aβ deposition, NFT formation, and neurodegeneration may be consequences of mitochondrial malfunctioning 65. The overexpression of APP and treatment with amyloid-β-derived diffusible ligands induce mitochondrial fragmentation and abnormal distribution without cell death 66, 67, which suggests that Aβ-induced abnormal mitochondrial dynamics may play a role in the early pathogenesis of AD 68. Aβ induces axonal and dendritic swelling and decreases axonal transport in hippocampal neurons 69, which contribute to an altered mitochondrial distribution. Excessive mitochondrial fission correlates with increased reactive oxygen species (ROS) production 66, 67, 70, which leads to the progression of extensive macromolecular oxidative damage and amyloid lesions caused by ROS 71. Intracellular Aβ interacts with 3-hydroxyacyl-CoA dehydrogenase type-2 (HSD17B10, also known as ABAD), and this interaction promotes mitochondrial dysfunction and ROS leakage 72. Another protein localized at the outer mitochondrial membrane, dynamin-1-like protein Drp1 (DNM1L), interacts with Aβ and phosphorylated tau, which likely leads to excessive mitochondrial fragmentation and neuronal damage 73. Tau is a substrate for mitochondrial caspase-3 *in vitro* 41, 74, which contributes to mitochondrial fragmentation in AD brains. Caspase-cleaved tau and tau truncated at Asp421, which mimics the product obtained with caspase cleavage (T4C3), induce mitochondrial fragmentation and mitochondrial fission in a calcineurin-dependent manner 75. The expression of caspase-cleaved tau fragments in neurons impairs mitochondrial function, and this impairment leads to changes in the mitochondrial membrane potential, the dysregulation of mitochondrial calcium levels and mitochondrial integrity and an increase in mitochondrial O^2-^ 68, 75. The introduction of tau in mature hippocampal neurons results in the degeneration of synapses via perturbation of mitochondrial transport and ATP levels at the synapse 76. N-terminal tau fragments exert toxic effects on mitochondria and lead to mitochondrial dysfunction associated with impairments in oxidative phosphorylation by distorting the structure of the complex V enzyme and the level of adenine nucleotide translocator, which ultimately perturb the synthesis of ATP in mitochondria 77. The dysregulation of Ca^2+^ and its secondary processes directly affect tau phosphorylation, APP processing and lysosome function, and in turn, tau and Aβ further aggravate Ca^2+^ dyshomeostasis 78. Aβ stimulates neurons to cause a large amount of Ca^2+^ influx and intracellular Ca^2+^ overload then damage to mitochondria, which results in the impairment of neuronal productivity, activation of apoptosis-related proteins and factors and the initiation of apoptosis 79. The dysregulation of Ca^2+^ homeostasis further promotes mitochondrial dysfunction, impairments in synaptic transmission and plasticity and oxidative stress, which contribute to the age-related cognitive impairment. Aβ and tau exert deleterious effects on mitochondria, and these effects result in ROS accumulation, ATP synthesis and Ca^2+^ dysregulation 80 (Figure 2).

## Joint action of Aβ and tau in nonneuronal cells

### Combined effects of Aβ and tau on microglia in AD

**Figure 3:**
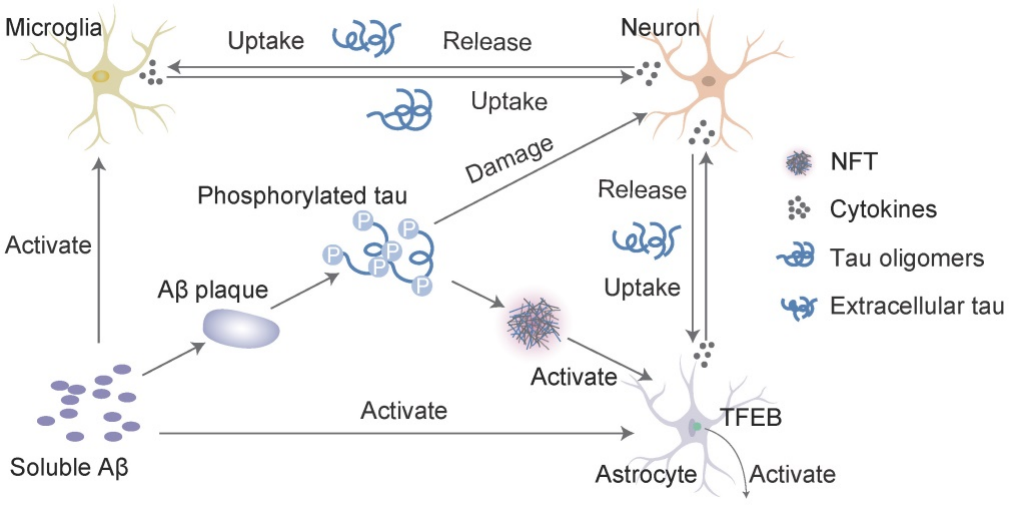
** Interaction of Aβ and tau with microglia and astrocytes.** In response to Aβ or tau, microglia and astrocytes are converted into a reactive state, which triggers the inflammatory cascade. These cells reciprocally activate each other via this cascade, which leads to neuronal injury. Inflammatory cytokines (such as interleukin-1β and TNF-α) induce the neuronal release of tau, and activated microglia take up extracellular tau. Cytokines also accelerate tau phosphorylation to ultimately induce the formation of neurofibrillary tangles (NFTs). Transcription factor EB (TFEB) activates astrocytes to take up extracellular tau. This process consists of a cycle of Aβ deposition, tau phosphorylation, release and uptake, and the roles of cytokines to ultimately lead to cell death and the NFT-mediated exacerbation of neurodegenerative changes.

Microglial activation is a key neuropathological feature of AD, and clinical studies found reduced levels of glial cell activation in more benign cases of AD 81. Soluble Aβ and subsequent plaques trigger microglial activation and the release of proinflammatory cytokines, such as interleukin-1β and tumour necrosis factor-α (TNF-α), ROS and reactive nitrogen species 82. The neuroinflammatory reaction in the brain caused by activated glial cells primarily occurs around capillary sites where Aβ is deposited 83. The expression of mutant tau also drives microglial activation, even prior to tangle formation 84 (Figure 3). Young APP/PS1-rTg4510 mice without plaques and tangles exhibit a synergistically enhanced tau aggregation phenotype, which may be an early consequence of microglial changes. This result directly implicate microglial changes in the progression of tau pathology 85. Microglial depletion protected against the propagation of tau pathology from the EC to the dentate gyrus (DG) in PS19 transgenic mice and rescued tau-dependent DG hypoexcitability 86. Glial tau pathology reportedly occurs in tauopathies 87 and AD 88. Microglia take up and decompose seed-competent tau 89, albeit ineffectively 90, and tau uptake may reciprocally lead to microglial activation. Activated microglia may be directly involved in the repackaging of tau into exosomes or indirectly involved in the enhancement of tau phosphorylation via proinflammatory cytokine signalling 85, 91. Microglia activated as a consequence of neuronal injury may also be related to the induction of neurodegeneration, which leads to tau hyperphosphorylation and aggregation 92. Enhanced microglial activation and accelerated onset and progression of tau pathology may be caused by the absence of the microglial fractalkine receptor CX3CR1 85, 91. Tau associated with exosomes and phosphorylated at Thr181 (AT270^+^ tau) was identified in human cerebrospinal fluid (CSF) samples from patients with AD 93. Therefore, microglia may phagocytose tau-containing neurons or synapses and secrete tau protein via exosomes 93, 94. During this process, microglia may facilitate the spread of tau pathology via exosomal vesicles. Variation in the TREM2 gene is a major risk factor for AD. TREM2 deletion and the secondary loss of plaque-associated microglia enhance the seeding and spreading of tau pathology, which suggests that TREM2 mitigates tau pathology by directing microglia to scavenge plaques 95. Microglia may also neutralize Aβ or toxic tau species to delay propagation and neurodegeneration. Briefly, activated microglia may play a role in Aβ toxicity and tau pathology and the formation of neuritic plaques and tau hyperphosphorylation, which contribute to microglial activation.

### Interaction of Aβ and tau with astrocytes in AD

Astrocytes are abundant in the brain and perform many functions in the central nervous system (CNS), such as modulating synaptic formation, maintaining neuronal homeostasis via metabolic support 96, and comprising part of the blood-brain barrier (BBB) 97. Astrocytes may be activated in the preclinical stages of AD 98. One of the earliest neuropathological changes in AD is the accumulation of astrocytes at sites of Aβ deposition 99. Activated astrocytes surround amyloid plaques and NFTs, which are the neuropathological hallmarks of advanced AD 100. Aβ deposition and the NFTs formed by tau hyperphosphorylation may promote the activation and accumulation of astrocytes. Reactive astrocytes release proinflammatory mediators and cytotoxic molecules in neuroinflammation, and these effects exacerbate the pathology of AD 101. However, astrocytes also express genes involved in phagocytosis, which may attenuate the pathology via the uptake and clearance of protein aggregates 102. This phagocytic capacity was demonstrated for Aβ 103, 104. Insights from animal models indicate that impairing astrocyte activation via the ablation of glial fibrillary acidic protein (GFAP) and vimentin resulted in an increased plaque load 105. Aβ is taken up and trafficked to lysosomes for degradation in astrocytes 103. The impairment of lysosome function with ageing 106 or the loss of presenilin is likely the underlying mechanism for the accumulation of Aβ and phagocytosed amyloid material within astrocytes 107, which may be the key to promoting amyloid plaque progression 100. Transcription factor EB (TFEB) plays an important role in this process. Astrocytic TFEB expression attenuates amyloid plaque accumulation by enhancing Aβ uptake from the interstitial fluid (ISF) and facilitating clearance via lysosomal degradation in the brain. TFEB directly stimulates astrocytes to degrade Aβ, which reduces the pathogenesis of amyloid plaques (Figure 3). In a transgenic model of tau spreading, astrocytes took up hyperphosphorylated tau as the synapses degenerated 108. The accumulation of abnormally phosphorylated tau in astrocytes is named ageing-related tau astrogliopathy (ARTAG) 109. ARTAG may play a role in mediating the formation of pathological tau inclusions in astrocytes or indicate astrocyte involvement in the early stages of transsynaptic tau spreading 109. TFEB in astroglia stimulated the uptake and clearance of aberrant extracellular tau, which prevented the neuronal spreading of tau pathology in a mouse model of tauopathy 110. In conclusion, Aβ and tau may share a common site of action in astrocytes, which promotes the expression of TFEB activated by Aβ deposition and the over-deposition of hyperphosphorylated tau. Damage to astrocytes reduces TFEB expression, which results in reduced clearance and increased aggregation of Aβ and tau. In contrast, Aβ over-deposition and tau hyperphosphorylation accelerate astrocyte injury by releasing neuroinflammatory cytokines. However, excessive damage to astrocytes worsens the inadequate removal of Aβ and tau, which further aggravates self-injury and the progression of AD.

Aβ and tau may be involved in damage to the BBB, endothelial cells, and pericytes during the pathological process of AD. Aβ and tau activate microglia and astrocytes. Activated microglia disrupts chemokine and cytokine secretion from the BBB 111. Microglia-mediated BBB alterations in AD are also reflected by the destruction of pericytes and basement membrane (BM) by microglia. Activated microglia also promotes pericyte apoptosis *in vitro* via the upregulation of NADPH oxidase in pericytes 112. Activated astrocytes secrete vascular permeability factors, such as vascular endothelial growth factors (VEGFs), nitric oxide (NO) 113, and endothelin (ET) 114, which aggravate the destruction of the BBB. Aβ and tau may exert indirect or direct effects on BBB components. Currently available data are insufficient. Therefore, the specific mechanism must be further examined, and a further understanding may help identify new methods for the treatment and prevention of AD.

### Aβ plaques facilitate neuritic plaque tau aggregation and propagation

Widespread deposition of Aβ plaques in the neocortex and a hierarchically organized pattern of NFTs (composed largely of tau aggregates) in limbic and cortical association areas are the neuropathological hallmarks of AD 5. Aβ and p-τ pathology spread throughout the brain in a hierarchical pattern. However, prior to NFT formation in the transentorhinal region, an accumulation of p-τ is observed in the locus coeruleus, raphenuclei, substantia nigra, dorsal nucleus of the vagus nerve, and basal nucleus of Meynert 115-117. Aβ plaques and NFTs are found in neocortical brain regions after a certain stage 118, 119. This finding suggests that neurodegenerative lesions spread or propagate from one brain region to another 120. Nevertheless, the mechanisms of Aβ and tau pathology interactions and whether other proteins are involved in this process are not clear and should be the focus of our attention and thinking.

A recent study showed that cellular prion protein (PrPC) was a receptor for toxic Aβ species and α-synuclein oligomers 121. PrPC occurs in the neuropil 122, 123, and it was detected in amyloid plaques and neurons in patients with AD 124, 125. PrPC is enriched in postsynaptic densities, which leads to Fyn kinase activation, and activated Fyn kinase phosphorylates the GluN2B subunit of NMDA receptors 126 and interacts with the phosphorylation of tau 127, 128. Aβ oligomers initiate PrPC-Fyn-related phosphorylation of tau 129. PrPC may be directly or indirectly involved in the interplay between Aβ and p-τ pathology propagation. Soluble p-τ may be released at synapses 130 and may be present in the extracellular space, which creates the conditions for the binding of soluble p-τ to PrPC. Aβ and PrPC combine with an interaction partner to activate Fyn 126, 131, which increases the levels of p-τ via Pyk2-related phosphorylation of tau 132, 133. Tau appears to spread into neocortical regions almost solely in people with coexistent Aβ pathology 134, 135. Tau-knockout neurons are resistant to neuritic degeneration induced by synthetic or human-derived Aβ species, and tau overexpression exacerbates Aβ-induced damage 136. During this process, the binding of Aβ to PrPC 137, Fyn activation, and τ-protein phosphorylation may provide alternative explanations for the finding that Aβ plaques accelerate tau phosphorylation propagation via a PrPC-related mechanism, which may encourage the deposition of tau in areas of the brain where Aβ aggregates. These correlations at the lesion site again demonstrate direct and indirect interactions between Aβ plaques and tau aggregation.

### Relationships between Aβ, tau and cognition

Aβ and p-tau interact to cause neuronal loss and synaptic damage, which lead to cognitive decline in patients with AD 68, 138. The accumulation of plaques and tangles induce changes in the behavioural symptoms of patients with AD, which are a direct consequence of the damage and destruction of synapses that mediate memory and cognition 53. Previous longitudinal PET studies tracked Aβ 139, 140 and tau 141, 142 accumulation. Clinical studies observed greater cognitive decline in clinically healthy older individuals with abnormalities of CSF Aβ and phospho-tau 143, 144. PET and CSF data indicate that synergy between Aβ and tau is associated with brain dysfunction 145, 146 and cognitive decline 144, 147. Aβ and tau in the inferior temporal neocortex interact and potentiate tauopathy and cognitive decline. One clinical study indicated that memory progressively declined in individuals with posterior cingulate hypometabolism, which is the region most strongly linked to the tau-Aβ interaction 145. During AD progression, Aβ and tau proteins are readily excreted from the brain into the peripheral blood through disruption of the BBB and receptor-mediated mechanisms 148, 149. Aβ and tau are detected in plasma using highly sensitive and accurate analytical techniques. Single-molecule array (SIMOA) and xMAP technology determined that Aβ42 levels, which are positively correlated with CSF Aβ42 levels but negatively correlated with the CSF t-tau levels, were higher at the mild cognitive impairment (MCI) stage than the AD stage, and the t-tau levels were positively associated with the burden of brain tau deposition on tau PET across the AD spectrum; i.e., the Aβ42 levels are elevated during the MCI stage and reach a plateau prior to the demented stage, whereas the t-tau levels increased with AD-associated tau pathology 150, 151. These findings are also consistent with laboratory findings, which suggested that tau was likely the primary mechanism of Aβ-related neurotoxicity 136 and previous work identifying other imaging markers of neurodegeneration 145, 152, 153, which indicated that Aβ pathology alone may be insufficient to drive imminent cognitive decline 154, 155. Previous autopsy studies 156 also support the notion that Aβ pathology precedes and accelerates neocortical tau pathology, which together precipitate cognitive decline. These findings also explain why the clinical treatment of Aβ has not yielded satisfactory results. Tau and amyloid pathology may begin independently, but the spread of tau beyond the mesial temporal lobe is associated with and may be dependent on amyloid accumulation 157. Therefore, higher plasma Aβ42 and t-tau levels at the MCI stage are predictors of greater risk for the development of cognitive decline at the predementia stage of AD. The combination of the two plasma biomarkers with other markers may help identify subjects with MCI who are at risk for developing AD, which may be beneficial for the administration of early and preventative pharmacological interventions.

## Conclusions and future directions

Aβ and tau proteins play well-established roles in AD and form the two hallmark pathologies that are visible in postmortem AD brains as amyloid plaques and NFTs, respectively. Although these two proteins play important roles in the pathogenesis of AD, treatments that target Aβ or tau alone have not achieved good clinical results 6-11. Related experimental and clinical studies were performed, which support the hypothesis that the interplay between Aβ and tau amplifies the toxic effects of each protein 13-15, 27, 47, 48. Some efforts have been made to interfere with the interaction between Aβ and tau protein, such as studies on GSK-3β and CDK-5 inhibitors 31-33. However, the exact clinical efficacy is not certain 34. The p38/JNK MAPK pathway also mediates cortical neuronal apoptosis 158, and ERK is related to memory and learning 159. Inhibition of ERK1/2, p38 and JNK kinases may be efficacious for the treatment of AD through attenuation of Aβ-induced neurotoxicity via activation of the Erk1/2, p38 and JNK pathways 158, 159. However, the pharmacological inhibition of ERK1/2 in mice and SH-SY5Y cells did not reduce the basal levels of phospho-tau or hypothermia-induced tau hyperphosphorylation 160, 161. This result may be due to the multiple mechanisms involved in the interaction between Aβ and tau protein, and inhibitors cannot simultaneously inhibit the pathways of multiple targets. Clinical drugs for the treatment of AD primarily include cholinesterase inhibitors (donepezil, rivastigmine, and galantamine), which are effective for cognition in mild-to-moderate AD, and NMDAR antagonists (memantine), which are effective for moderate-to-severe AD 162. Combination therapy (cholinesterase inhibitors and memantine) may be beneficial for moderate-to-severe dementia 153. However, neither cholinesterase inhibitors nor memantine are effective in patients with mild cognitive impairment 162. AD is a multifactorial disease that likely is the result of different interactions of genomic, epigenetic, interatomic, and environmental aspects 163, 164. Therefore, a multidisciplinary approach is needed. We need to focus not only on pharmacological therapy but also on the complex biopsychosocial aspects of caring for patients with AD, such as by developing psychological interventions. Moreover, the development of new drugs that can treat AD via multiple pathways should be further examined.

## Figures and Tables

**Table 1:** Sites on tau phosphorylated by different kinases

Phosphorylation stage	Kinase	Phosphorylation sites	Whether Aβ is involved	References
Prephosphorylation	A-kinase	Ser262, Ser293, Ser305, Ser324, Ser356	No	18, 19, 24
	C-kinase	Ser305	No	24
	CaM kinase II	Ser416/Ser262	No	19, 24
	CDK-5	Ser195, Ser202, Thr231, Ser235, Ser396, Ser404	Yes	19, 23, 24, 28
Phosphorylation	GSK-3β	Ser199*, Ser202, Thr231*, Ser235, Ser262, Ser396*, Ser404*	Yes	19, 23, 24, 28
	MAPK	Thr181, Ser202*, Thr205*, Ser396*, Ser404*, Ser422, Ser199*, Thr50*	Yes	36-38
Prephosphorylation + Phosphorylation	A-kinase + GSK-3β	Ser199*, Ser202*, Thr231, Ser235, Ser262, Ser396*, Ser404*	Yes	24, 25
	C-kinase + GSK-3β	Ser199, Ser202, Thr231*, Ser235*, Ser262, Ser396*, Ser404^d^*	Yes	24, 25
	CaM kinase II + GSK-3β	Ser199*, Ser202*, Thr231*, Ser235*, Ser262*, Ser396*, Ser404*	Yes	24, 25
	CDK-5 + GSK-3β	Ser199, Ser202, Thr231*, Ser235*, Ser262, Ser396, Ser404^d^	Yes	23-26

Note: * Phosphorylation of these sites is significantly enhanced.

## Abbreviations

Aβ: amyloid‑β
AD: Alzheimer's disease
NFTs: neurofibrillary tangles
MT: microtubule
CDK-5: cyclin-dependent kinase-5
GSK-3β: glycogen synthase kinase-3β
MAPKs: mitogen-activated protein kinases
CASP3: caspase-3
PHF: paired helical filament
PD: projection domain
NMDA: N-methyl-D-aspartate
NMDAR: N-methyl-D-aspartic acid receptor
NR2: NR subunit 2
EC: entorhinal cortex
PSD-95: postsynaptic density protein 95
APP: amyloid precursor protein
ROS: reactive oxygen species
DNM1L: dynamin-1-like protein Drp1
TNF-α: tumour necrosis factor-α
DG: dentate gyrus
TREM2: triggering receptor expressed on myeloid cells 2
CNS: central nervous system
BBB: blood-brain barrier
GFAP: glial fibrillary acidic protein
CSF: cerebrospinal fluid
TFEB: transcription factor EB
ISF: interstitial fluid
ARTAG: ageing-related tau astrogliopathy
BM: basement-membrane
NADPH: nicotinamide adenine dinucleotide 2'-phosphate reduced tetrasodium salt
NADH: nicotinamide adenine dinucleotide
β-APP: amyloid-β precursor protein
VEGFs: vascular endothelial growth factors
NO: nitric oxide
ET: endothelin
PrPC: prion protein
PET: positron emission tomography
SIMOA: single-molecule array
MCI: mild cognitive impairment
